# New York Odontological Society

**Published:** 1884-08

**Authors:** 


### NEW YORK ODONTOLOGICAL SOCIETY.

A regular monthly meeting of this society was held June 17th, at the residence of Dr. E. A. Bogue, 29 E. 20th Street, the President Dr. W. Jarvie, Jr., in the chair.

*Dr. C. F. Ives*—Spoke of a case of pyorrhea alveolaris, that had yielded to treatment beyond expectation, showing marked evidences of cure, yet he did not feel sure that the patient would not again be attacked by this annoying disease. The doctor also exhibited a small glass syringe, very neatly constructed, for injecting remedial agents into diseased alveolar territories.

*Dr. N. W. Kingsley*—Referred to a paper read before the society two months previously, by Dr. Dodge, on the tendency of some amalgam fillings to assume a spherical form after becoming hard. Conceding that *some* fillings of alloy might shrink or change shape, Dr. Kingsley felt sure that the amalgam he was using would neither shrink nor expand. He attributed the changes spoken of as "recession of plugs from cavity walls," to improperly preparing cavities, or leaving the edges untrimmed and frail.

*Dr. J. W. Clowes*—Proclaimed enthusiastically the virtues of amalgam. He admitted that some of the alloys in market were bad, but that very many were good. He believed that failures resulting from its use were due to improper manipulation, as comparatively few knew how to use it. He would not give much for an amalgam filling made by a dentist who could not make a good gold filling. He had seen many cases where it had been used by dentists of high standing, but whose operations were miserably performed. His own fillings remained firm and did not change shape. He urged dentists to learn to use amalgam, that the difficulties referred to might disappear.

*Dr. J. Smith Dodge, Jr.*—Did not wish it understood that the paper he read condemned the use of amalgam, for he often employed this material in his own practice; neither did he state that *all* alloy fillings changed shape. It was nevertheless a fact that many *did* shrink and bulge out, and the object of his paper was to give what he considered the most reasonable theory for such changes. He did not believe that what was termed "shrinkage" was due to a neglect to trim cavity margins properly, for such changes are found in fillings on grinding surfaces of molars, where the cavity-walls are thick and strong. He had a tooth in his possession which he filled after extraction, with strong and well-trimmed walls, and although smoothly finished and burnished after it became hard, the filling had since receded from the cavity-margin and bulged out at the top. "It is somewhat curious" added the doctor, "that the failures we witness are usually from operations performed in the offices of our neighbors, and not in our own."

*Dr. A. H. Brockway*—Could say, from experience and observation that many amalgam fillings not only "bulged" but "budged." It was this *budging* that troubled him, and he was interested to know the cause of such changes.

*Dr. C. E. Francis*—Remarked that the interest he took in Dr. Dodge's paper had led him to notice more particularly of late the appearance and condition of amalgam fillings that came under his observation. He had in this time seen a number of defective ones, some of which he had removed and substituted gold. Several were on grinding surfaces of molars, where the enamel was hard and the cavity-walls thick and firm.

*Dr. W. A. Bronson*—Read a paper in which he discussed the relative merits of the various forms of gold-foil for filling teeth. He objected to the practice of massing foil into balls, or rolling it into coils, on account of the difficulty of conforming them to the shapes of cavities. He considered ribbons of folded foil preferable, as slight pressure would force the air from between the folds and cause the flat surfaces to readily unite. For many cases, especially large approximal cavities, irregular and extending far into their cervical borders, he preferred cylinders, believing they can be more securely packed and that they make more reliable fillings. In some instances he commences with very large cylinders of soft tin foil.

*Dr. J. B. Rich*—Did not favor the use of cylinders, nor did he consider this form of foil capable of being reduced to a homogeneous mass. Upon pressure each cylinder becomes a flattened ellipse, the folds or layers increasing in number, and the cylinder in thickness as the centre is approached. Viewed in a scientific light, he declared it an impossibility to make a perfectly solid filling with cylinders. He asserted that ribbons of gold foil could be more perfectly adapted to cavity-walls, and would make the most compact fillings.

*Dr. W. H. Dwinelle*—Disapproved the use of cylinders, for reasons already stated by Dr. Rich. He preferred folds or ribbons. Dr. Dwinelle also referred to his success with prepared, or crystal gold.

*Dr. E. A. Bogue*—Presented two gold fillings of exactly the same shape and size, which he formed in a steel matrix—one from strips of heavy foil, and the other from cylinders. The former required much over an hour to consolidate ; the latter was produced in less than twenty minutes. He favored the use of cylinders for many cases, believing they can be manipulated into excellent fillings in the least time. The saving of time, and consequently money, is with many patients a matter worthy of consideration.

